# Assessment of cerebrovascular reactivity during major depression and after remission of disease

**DOI:** 10.4103/0972-2327.61278

**Published:** 2010

**Authors:** Alireza Vakilian, Farhad Iranmanesh

**Affiliations:** Department of Neurology, Rafsanjan University of Medical Sciences, Rafsanjan, Iran

**Keywords:** Cerebrovascular reactivity, transcranial Doppler ultrasound, depression

## Abstract

**Background:**

There are a growing number of studies suggesting that depression may increase the risk of stroke. Impaired autoregulation of vascular tone may contribute to a higher risk of developing cerebrovascular diseases. Cerebrovascular reactivity (CVR) reflects the compensatory dilatory capacity of cerebral arterioles to a dilatory stimulus and is an important mechanism that ensures constant cerebral blood flow. There is a hypothesis that CVR is reduced in major depression, which would explain the association between depression and stroke.

**Objectives:**

The aim of this study was to investigate the effect of depression on CVR in cerebral vessels by comparing CVR during the depression phase with that during remission.

**Material and Methods:**

Using the apnea test, we assessed CVR in 16 patients with unipolar depression during disease and after remission of disease by calculating the increase in cerebral blood flow velocity after breath-holding (the apnea test). Blood flow velocities were measured by transcranial Doppler ultrasound (TCD).

**Results:**

CVR was significantly reduced in the depression phase in comparison to that in the remission phase. However, this change was not seen in all the patients.

**Conclusion:**

CVR was reduced in most of the depressed patients. The decreased CVR, as indicated by the changes in peak systolic velocity (PSV) and mean flow velocity (MFV) of the middle cerebral artery, in depressed patients was more marked on the right side, which could point to a vascular basis for some kinds of depression. We recommend that other studies, with larger samples, be done; future studies should assess whether the changes in the CVR varies with the severity and type of depression.

## Introduction

There is growing evidence that depression may increase the risk of stroke. Some large prospective epidemiologic studies have found a higher rate of cerebral infarction among people who suffer episodes of depression, and the higher risk remained statistically significant even after adjustment for confounding factors such as body mass index, smoking habits, diabetes, cholesterol level, gender, blood pressure, alcohol consumption, physical activity, race, and education.[1–5] Thus, depression may be a risk factor for stroke, independent of traditional cardiovascular risk factors. However, the pathophysiologic mechanisms leading to this association between depression and stroke are not well understood. There are a number of plausible mechanisms that could explain why depression may increase the risk of cerebrovascular disease, the most important ones being sympathoadrenal hyperactivity, platelet activation, increase in inflammatory cytokines, and increased risk of arrhythmia.[6]

The main factors modulating cerebral blood flow velocity are blood viscosity and vascular tone. Impaired autoregulation of vascular tone may contribute to a higher risk of developing cerebrovascular disease. Cerebrovascular reactivity (CVR) reflects the capacity of cerebral arterioles to respond to a dilatory stimulus; this mechanism plays an important role in maintaining a constant cerebral blood flow. CVR seems to be gaining importance as a prognostic factor for stroke risk. CVR has been determined by calculating the difference between maximal blood flow velocity during rest and that after stimulation with acetazolamide.[7] Previous studies have shown a decreased vasodilatory capacity under various circumstances, for example, in subjects with long-term insulin-dependent diabetes[8] or non-controlled hypertension. Moreover, impaired CVR in young hypertensive subjects appears to be improved after the initiation of antihypertensive treatment.[9] Cerebral autoregulation is impaired in acute conditions such as hypertensive encephalopathy, cerebral infarction, trauma, and intracranial infection. In the absence of major arterial stenosis, impaired CVR is associated with a higher risk of stroke.[10,11] In another study, male gender, age, and the presence of lacunar infarction on magnetic resonance imaging (MRI) were found to be significantly and independently associated with reduction of CVR.[12]

In the apnea test, which is one of the methods for assessing CVR, the increase in the cerebral blood flow per time period (quantitative examination) following breath-holding is measured. This is an easy way to measure CVR in cooperative patients. Recent studies using this method compared depressive patients with normal controls, but some confounding factors are likely to be present in such an assessment.[13] To avoid this, we compared CVR changes following apnea in depressive patients with the changes seen when in remission. Since the patients serve as their own controls, many confounders were removed.

The aim of this study was to investigate the effect of depression on CVR in cerebral vessels by comparing reactivity during the depression phase with that during remission.

## Materials and Methods

In this prospective cohort study we included 16 depressed patients; each patient was examined twice: once during the depressive illness and then again after remission. According to our inclusion criteria the subject had to have unipolar depression, as defined by DSM-IV criteria, the method of patient selection was based on an questionnaire (structured clinical interview for DSM-IV, Axis 1 Disorder) that was prepared for unipolar depression diagnosis in DSM-IV, Axis 1 disorder;.[14] and be between 18 and 45 years of age. Pregnancy; presence of any vascular risk factor (e.g., diabetes, hypertension, smoking); a history of using drugs that have action on the vascular system (e.g., betablockers, calcium channel blockers); neurologic disease (any kind of stroke); cardiac disease (myocardial infarction), vascular diseases; or psychiatric illness led to exclusion from the sample. Smokers were also excluded from our study; all our study subjects were non-smokers who had abstained from smoking for at least the past 15 years or had never smoked at all.

Skilled psychiatrists chose the cases, making the diagnosis strictly according to the clinical scales mentioned above. Most of the cases included in this study were being admitted to hospital for treatment for the first time.

All patients underwent careful neurologic and cardiologic examination, as well as blood chemistry analysis. A detailed clinical history was taken, with particular attention to vascular risk factors. These risk factors included cardiac arrhythmia,coronary heart disease, hypertension, diabetes mellitus,hypercholesterolemia, and hypertriglyceridemia. Hypertension was defined as either systolic blood pressure values greater than 130 mm Hg and/or diastolic blood pressure greater than 90 mm Hg on two of six different determinations on three different days or history of use of antihypertensive medication.Diabetes mellitus was defined as fasting glucose levels greater than 5.7 mmol/l or history of treatment for the condition.Hypercholesterolemia was defined as cholesterol level greater than 5.2 mmol/l or high-density lipoprotein (HDL) lower than 3.9 mmol/l or history of treatment for the condition; and hypertriglyceridemia was defined as triglyceride level more than 1.7 mmol/l or history of being treated for the condition.

Most of the patients (14 cases) were not treated with any drugs while admitted to our hospital. Two of the patients were already taking an antidepressant (fluvoxamine, fluoxetine, or sertraline) and had suffered a relapse. Depression was measured using the Hamilton Depression Scale.[15]

All participants gave written informed consent. The study was approved by ethics committee of Rafsanjan University of Medical Sciences.

Forty-two patients were examined initially. Twenty-six were excluded for various reasons: four patients were cigarette smokers, four were not cooperative, 14 patients had used drugs recently, and four patients had other risk factors (e.g.,hypertension, hyperlipidemia).^[26]^ Finally, therefore, 16 patients remained in our study sample.

CVR investigation was performed by the same physician on all patients at the same time of the day, i.e., between 10 and 12 AM. All patients were asked to abstain from caffeine for at least 2 h before the examination.

To exclude the presence of any intracranial stenosis that might interfere with the measuring of CVR, a complete Doppler examination of the anterior, middle, and posterior cerebral arteries; internal and external carotid arteries; ophthalmic arteries; carotid siphons; and the basilar and vertebral arteries was performed in all patients.

Four indices: mean flow velocity (MFV), peak systolic velocity (PSV), end-diastolic velocity (EDV), and pulsatility index (PI) of all these vessels were checked by means of a Nicolet TC-22 Legend TCD instrument. For flow detection, a 4-MHz transducer was used for the extracranial vessels and a 2-MHz transducer for the intracranial vessels. The depth of study was matched with site that have highest velocity in each intracranial artery. All velocities were calculated in centimeters per second.

The investigation was performed in a quiet room, with the patient lying in a comfortable supine position without any movement and noise. At the first examination in the disease period, the four indices in both the right and left middle cerebral arteries (MCAs) were first recorded. Later the same day, the indices were recorded again, but this time after 30 s breath-holding (the apnea test). Respiratory and cardiac monitoring was done during the examination was performed. Although this method of breath-holding (the apnea test) is generally considered to be less accurate than the acetazolamide test, we preferred to do this because it requires little intervention and is very simple to perform. Four patients were excluded from study because they did not cooperate during the apnea test.Non-cooperation by the patient during the apnea test can be a significant problem in the disease phase; we assumed that the patients who cooperated in the disease phase would also do so in their remission phase. Four to six months later, after the patients had been treated by psychiatrists and were free of depression, the MCA velocities and PIs were again recorded before and after breath-holding. In both phases, CVR was determined by calculating the difference between the maximal velocities before and after stimulation (breath-holding). Improvement in CVR was defined as increasing the difference between velocities before and after breath-holding as respected in normal cases. The highest velocity that was recorded in breath-holding phase, was chosen as respected velocity in that phase.

The comparison of the results obtained in the two phases was done after collection of data.

### Statistical analysis

The sample size was calculated on the basis of the results from other studies. In the study by Neu *et al*.[13] a 20% reduction in CVR was found in depressed patients in comparison to normal controls, with α = 0.05 and power = 80%. According to these results we calculated that we would require a sample size 16, with each participant acting as his/her own control in the remission phase. We used the paired t-test to compare the results obtained in the two phases.

## Results

In this study we included 16 unipolar depressed patients and investigated their CVR at two separate periods: during depression and during remission. Clinical data are shown in Table 1.

**Table 1:** Clinical, demographic, and hemodynamic parameters of depressed patients

Variables	Value
Age (mean ± SD)	32.06±6.05
Female sex (%)	72
Height (cm) (mean ± SD)	160.45±12.23
Weight (kg) (mean ± SD)	55.32±8.03
Systolic blood pressure (mean ± SD)	110.06±9.40
Diastolic blood pressure (mean ± SD)	78.32±8.5
Heart rate (mean/SD)	80±7.54
MFV in LT* MCA (cm/s)	54.31
MFV in RT** MCA (cm/s)	52.84

* LT: Left

** RT: right

We compared patients’ response to the apnea test during their depressive phase with their response 4–6 months later when they were in remission. Before carrying out the test we checked all the brain vessels and ensured that there were no stenoses or abnormalities in flow velocities. The mean of all flow velocities of the vessels are shown in Table 2.

**Table 2:** Mean (SD) of flow velocities in patients in all intracranial and extracranial vessels

Artery	MFV		PSV		EDV		PI	
				
	L	R	L	R	L	R	L	R
Internal carotid artery	65.69 (14.45)	63.81 (13.65)	24.65 (8.2)	22.20 (6.87)	35.8 (6.77)	35.96 (7.83)	1.17 (0.20)	1.19 (0.29)
Middle cerebral artery	79.84 (25.07)	83.01 (21.49)	37.81 (12.27)	39.99 (9.68)	54.31 (16.10)	56.01 (15.87)	0.79 (0.13)	0.80 (0.09)
Anterior cerebral artery	69.03 (14.47)	70.61 (14.62)	32.80 (8.23)	32.64 (8.21)	46.03 (10.25)	45.83 (10.89)	0.84 (0.11)	0.86 (0.09)
Posterior cerebral artery	51.05 (10.82)	50.23 (11.21)	23.17 (8.46)	23.53 (10.49)	34.20 (8.33)	32.99 (11.21)	0.91 (0.15)	0.95 (0.25)
Vertebral artery	53.92 (17.97)	49.85 (14.63)	26.11 (9.48)	24.20 (7.99)	35.57 (11.57)	33.29 (9.55)	0.87 (0.21)	0.85 (0.24)
Basilar artery	60.6 (10.14)		29.18 (8.97)		40.35 (10.49)		0.82 (0.25)	

PSV: Peak systolic velocity; EDV: end-diastolic velocity; MFV: mean flow velocity; PI: pulsatility index

After initially checking the flow velocities in all the vessels, the focus of the study was largely on the right and left MCAs. We compared the post-apnea changes in the MCA flow velocities in the disease and the remission phases. In all patients the mean flow velocity on the left side was 54.31 cm/s and after apnea it was 66.22 cm/s. On the right side, it was initially 56.01 cm/s and after apnea it was 69.41 cm/s. In the remission phase, the mean flow velocity of the left MCA was initially 53.50 cm/s and post apnea it was 67.61 cm/s. On the right side,the mean flow velocity was 52.48 cm/s and post apnea it was 71.36 cm/s. All of the measured velocities are shown in Table 3.The difference between mean flow velocities of the right MCA in the disease phase was 13.4 (69.41-56.01) and that in the remission phase was 18.88 (71.36-52.48); the difference was statistically significant (i.e., there was a significantly lower CVR in the disease phase). With regard to the PSV in the right MCA, the difference in the disease phase was 14.75 (97.76-83.01), while in the remission phase it was 24.54 (101.83–77.29); this difference was statistically significant. In the left MCA, in the disease phase, the difference between pre-apnea and post-apnea PI was 0.05, while in the remission phase it was 0.13; this too was a statistically significant difference.

**Table 3:** Flow velocities in middle cerebral arteries (MCA) in disease and remission phase, before and after apnea test

		MFV		PSV		EDV		PI	
					
		preAp	postAp	preAp	postAp	preAp	postAp	preAp	postAp
LT MCA	Disease phase	54.31	66.22	79.84	95.04	37.81	48.82	0.69	0.74*1
	Remission phase	53.50	67.61	78.04	93.33	36.55	52.14	0.85	0.72
RT MCA	Disease phase	56.01	69.41*2	83.01	97.76*3	39.99	50.13	0.80	0.76
	Recovery phase	52.48	71.36	77.29	101.83	37.10	51.92	0.81	0.74

Pre-apnea (preAp), post-apnea (postAp),

*1 Significant results LT MCA, PI differences (P value = 0.05)

*2 RT MCA, PSV differences (P value = 0.035)

*3 RT MCA,MFV differences (P value = 0.012).

None of the patients in our study had abnormal CVR in the remission phase; i.e., the apnea test did not cause significant lowering of velocity in any patient in the remission phase. Some patients had high CVR in the disease phase. Ten patients had low reactivity on the left side in the disease phase in comparison to remission stage. Eleven persons had low reactivity on the right side in the disease phase in comparison to the reactivity in the remission phase. As assessed by the changes in PSV, eight patients had low CVR in the depressive phase and only one patient had low CVR in the remission phase.

Significant results were found in some parts of study. In the right MCA, measurement of PSV revealed low CVR in the disease phase. In the disease phase the difference between pre-apnea and post-apnea PSVs was 14.75 cm/s and in the remission phase it was 24.64 cm/s; this difference was statistically significant (*P*=0.035). This is suggestive of decreased CVR during depression. Also, on the left side, the PI was significantly higher in the disease phase as compared to that in the remission phase (0.4 in disease phase *vs* 0.15 in remission phase; *P*=0.05). The other significant finding in our study was seen in the MFV on the right side; in the disease phase there was a 22% increase in MFV after apnea as compared to the pre-apnea flow velocity,while there was a 38% increase in the remission phase (*P* =0.012) [Table 3].

## Discussion

Our main finding is that patients suffering from a depressive episode have reduced CVR as judged by some flow velocities compared with the same patients after recovery from the disease. None of the patients had any vascular risk factors. An important finding was the absence of any reduction of CVR in the remission phase in any patient who had demonstrated reduced CVR in the disease phase.

Unlike Neu *et al*.[13] we took care to exclude smokers from our study population, so our findings are not influenced by any long-term effects of smoking on vascular reactivity. De Castro *et al*., in their study, ensured a 1-h abstinence from smoking prior to testing, but such measures can only help avoid the acute effects of smoking.[16] In a later study, they excluded current smokers from the study;[17] however, the long-term effects of smoking negatively influenced vascular reactivity in that study.

None of the patients in our study were taking any drugs at the time of the investigation. In the remission phase, we ensured that there was a 10–14 day gap between drug use and the reactivity study; it is unlikely that serum drug levels remained at the time of testing that could affect CVR. While there has been a study that indicated that fluoxetine increases cerebral blood flow in rats,[18] no research has been conducted in humans about the influence of antidepressants on cerebral blood flow.

We do not yet know whether the observed reduction in CVR, as indicated by the changes in some flow velocities, has clinical importance in terms of leading to a cerebrovascular insult in these patients; follow-up of these patients after remission would be interseting. Reduction of CVR in depression may have some role in increasing risk of stroke. Also there was heterogeneity of the CVR in the study population. Some of our patients had normal CVR. Thus, all depressed patients do not have reduced CVR. Although by excluding smokers and by avoiding the use of normal subjects as controls we avoided some of the possible confounders, our findings were not significantly different from that reported by Neu *et al*.[13] It is possible that the treatment of depression does not change the vascular properties of patients with depression so we could not find significant results and diffuse changes in all flow parameters as we expected.

This difference between depressed patients in CVR reduction may be related to severity of depression. A study with a larger sample, using careful assessment of cases with consideration of severity of disease and type of depression, should be able to throw light on this possibility. In this study, patients with other potential risk factors that may impact CVR were excluded; also, since the patients serve as their own controls the effects of the other risk factors on CVR will be automatically canceled out.

The other finding is that, as assessed by MFV and PSV, the decrease in CVR is predominently on the right side, which may be indicative of a vascular contribution to the pathogenesis of depression. On the other hand, the changes in PI show greater decrease in CVR on the left side.

We used the apnea test for assessing CVR in our patients instead of acetazolamide infusion. The former test does not require the use of any drug, but it needs patient cooperation; lack of cooperation can influence the results. We had to replace four patients with new cases due to lack of cooperation during study. There were some limitations in our study, such as the limited sample size, the highly selected sample, and the fact that uncooperative patients could not be subjected to the apnea test. Future studies should have larger samples and must be designed to examine the effect of severity and type of depression on CVR.
